# Rheumatism

**Published:** 1861-07

**Authors:** B. Woodward

**Affiliations:** Galesburg, Illinois


### Selections.

### RHEUMATISM.

By B. WOODWARD, M. D., of Galesburg, Illinois.

*From the Medical and Surgical Reporter.*

Having for the past fifteen years had a great many cases of rheumatism to treat, I have, with the deepest earnestness and patience, sought to ascertain its nature, but it is only within the past four years that my mind has become at all settled on the subject, and since I have adopted my treatment to my view of the pathology I have not had cause to regret it. Observation and my own course of reasoning have led me to the adoption of the hypothesis, that *all constitutional diseases, in which swelling, pain, or convulsions are prominent symptoms, are of purely nervous origin, and point unmistakably to a derangement of the nervous system as a whole, or in some of its more important parts.*

The nerves, so far as we know, are the only tissues in the body capable of suffering or transmitting pain; when it is manifested in other parts, as in muscle, bone, or fascia, it is only from the ramifications of nerve-fibre to these parts. This view does not exclude those lesions or affections of the nervous centres which are marked by paralysis, in which no pain exists, and unless the derangements are such as to produce paralysis, there is always pain or convulsions or both where there is nervous derangement. I shall only endeavor, in the present paper, to establish this view, so far as rheumatism is concerned.

It is not easy to conceive of a constitutional disease, caused by a depravation of either solids or fluids of the body, without functional lesion, and, hardly, without organic. Yet we do find this to be the case in diseases of the nerves, as in neuralgia, in which pain and sometimes increased action of the circulatory system are the only abnormal conditions. This condition also obtains in the majority of cases of rheumatism in the early stages, and until the nervous derangement has produced other abnormal actions.

Those who contend for the acid theory, or the abnormal amount of fibrine in the blood, as the cause of rheumatism, acknowledge that both the one or the other are often very rapidly formed and as rapidly disappear, but they have not proved that this is not caused by nervous action itself. Without calling on any to prove a negation, we may point to analagous cases to sustain ourselves.

Hydrophobia is a nervous disease of the purest type, and one of the products of the nervous irritation is the poison in the saliva. Where there is no constitutional disease, other than a temporary derangement of the nervous system, the urine becomes loaded with abnormal products, and all are familiar with the increased secretion of saliva, mucus and serum, as the case may be, arising from certain forms of nervous excitement, be the excitement of a healthy or unhealthy nature; and why may not an acid, or an increased quantity of fibrine, likewise depend upon nerve-irritation in rheumatism?

We have certain forms of rheumatism in which pain, paralysis, and atrophy are the only symptoms all through the history of the case, and these following each other in regular succession and, apparently, dependent on each other. * *

There is one form of so-called rheumatism which, if it do not depend upon nervous irritation, I do not know where to place it. I allude to rheumatism in a member after there has been fracture of a bone. This form is well known to every one who has treated many fractures, particularly of the femur and humerus, and I have often found it one of the most common and troublesome of the sequelæ; and, also, that it yields most readily to the action of the neurotic remedies. * * * * * * *

If rheumatism is not of nervous origin, why do we find woolen worn next to the skin to be one of the best prophylactics, and why are rubefacients so valuable? On the hypothesis that it is disease of the nerves, the usefulness of the treatment is easily explained, from the action on and through the cutaneous nerves. It may be urged that swelling of the parts, effusion, etc., militates against the view that the affection is nervous; but to my own mind it has no weight. Disease of the nerves does not and need not imply that the affection is uniform either in its nature or manifestations. It may, and doubtless is, governed, in great measure, by the nature of the tissue in which it appears, and also by the condition of the patient at the time of the attack. In one case it may result in atrophy of a part, while in another hypertrophy takes place.

There is no fact in physiology better established than that the nutrition of muscles is governed by nerve action, as well as their motion and sensibility. Irritation of nerve tissue may as well cause swelling and effusion as paralysis of a nerve cause atrophy and wasting of muscles or other tissue. The cutaneous nerves are readily affected by causes acting from the surface, and it is not improbable that the disease in question is often produced by external irritation, as by cold or wet. This view does not necessarily imply that the disease must be confined to the cutaneous nerves, for the nervous system is so much a unit that, if one part, even on the surface, is affected, the deeper seated may quickly become involved.

The irritation occasioned by a slight wound of a fibre of nerve in a distant part may induce tetanus, which is a disease of the nerve centres. Neuralgia, or painful disease of the nerves, as generally understood, is marked by pain of a peculiar character, and different from that of rheumatism; but this is no evidence that the rheumatism does not arise from nerve causes, for the same tissues are subject to different forms of disease. It is not possible, in the present state of our knowledge, to say what the peculiar condition of the nerves is, either in the one or the other disease, but pathologists may yet unravel the mystery. This, however, will not be done till we have found the right end of the skein.

If, together with the nature of nervous affections generally, and the nature of pain in rheumatism, we couple the evidence derived from therapeutics, it seems to me that we have as nearly-conclusive evidence, as the nature of the case will admit of, that all rheumatism is of a neurotic character; it may be from transient causes, or it may depend upon a diathesis. In the one case it will be acute, and yield more readily to treatment; while in the other it will be chronic, and difficult, if not impossible, to eradicate. We read of various kinds of rheumatism, and, among them, of the nervous; but the chief differences are made to depend upon the location. If it were an acid, or any other depravation of the blood, why is it not general instead of local? why does it change its place of attack? and why does it depend so much upon climatic and atmospheric causes for its return from time to time?

This paper is not intended to be declaratory, but suggestive, and these views are thrown out for examination. What, then, is the testimony of therapeutics? It may be given in a few words. Combine the testimony of all the best authors, and it is that "that class of remedies which act upon and through the nervous system are more to be relied on than any other, and sooner overcome the disease." * * * *

